# Pantoea Infections in the Neonatal Intensive Care Unit

**DOI:** 10.7759/cureus.13103

**Published:** 2021-02-03

**Authors:** Srinivasan Mani, Jayasree Nair

**Affiliations:** 1 Pediatrics/Neonatology, State University of New York, Buffalo, USA

**Keywords:** infant, intensive care units, neonatal, opportunistic infections, pantoea

## Abstract

*Pantoea *is a plant pathogen infrequently reported to cause opportunistic bloodstream infections. This gram-negative bacillus is a rare cause of hospital-acquired infections in newborn infants with high mortality. Since the creation of the new genus *Pantoea* in 1989, the evidence base available to neonatal health care providers is limited. Most of the available literature consists of case reports and case series. This review aims to consolidate the current reported literature on Pantoea infections, focusing on newborn infants and the neonatal intensive care unit (NICU). Prematurity and the associated relative immunocompromised state are major risk factors for hospital-acquired infections due to *Pantoea *in newborn infants. Recent advances in molecular biology have improved our understanding of the cross-kingdom pathogenesis exhibited by *Pantoea*. Respiratory symptoms and association with central venous lines are the most common clinical presentation of *Pantoea *bacteremia in newborn infants. Early institution of appropriate antibiotic therapy against this organism could be lifesaving. Therefore, it is critical for neonatologists to understand the clinical spectrum of *Pantoea *infections in NICUs.

## Introduction and background

Pantoea, a plant pathogen, is a rare cause of human infections [1]. Pantoea genus was previously included in the Enterobacter genus. This gram-negative bacillus had been reported to cause opportunistic bloodstream infections in neonatal ICUs [2]. Pantoea can cause human infections either as community-acquired infections, including occupational exposures or hospital-acquired infections. Various sites and organs can be affected by Pantoea, causing wound infections, synovitis, septic arthritis, osteomyelitis, bloodstream infections, peritonitis, cholelithiasis, endophthalmitis, endocarditis, dacryocystitis, urinary tract infection, meningitis, brain abscess, and respiratory tract infections. They can also cause allergy and hypersensitivity pneumonitis [3]. In children, especially newborn infants, the infections have a predilection for the respiratory tract, resulting in respiratory failure causing high mortality [4,5].

## Review

Methodology

We have assimilated research findings from our experience and an extensive review of the literature utilizing key terms in multiple databases, including PubMed, EMBASE, and Science Direct. We used the MeSH terms - "*Pantoea*," "infant OR newborn," "intensive care, neonatal" for our search. We included "*Enterobacter agglomerans*" instead of "*Pantoea*" in our search but did not yield any relevant additional studies to be included. We searched the database up until July 2020.

Epidemiology

Prevalence

*Pantoea* infections have been described to cause outbreaks in healthcare settings in the USA since 1970 [6]. The exact prevalence of the disease caused by this pathogen in the NICU is unknown because of the limited literature on this rare pathogen in this patient population. There are few case series reporting systemic infection with this organism in preterm neonates. From the available literature, the prevalence is estimated as low.

In a study of 6,383 patients between the 1st of January 1994 and 1st of June 2005 in the Netherlands, *Pantoea* colonization was reported in 125 patients (2%) without clustering [7]. Common sites of colonization include the trachea, urinary tract, and intestinal tract [8]. Another case series noted that five out of 1,665 newborn infants admitted to two different NICUs had nosocomial bloodstream infections due to *Pantoea* between January 2005 and December 2006 in Kuwait [2]. In a single-center study from Turkey that evaluated the clinical and microbiological characteristics of *Pantoea* infections in pediatric patients from 2000 - 2015, neonatal infections comprised 34.7% (eight out of 23 Pantoea isolates) [9].

Risk Factors

Prematurity and associated immature immune systems resulting in the relative immunocompromised state are the major risk factors for hospital-acquired infections due to *Pantoea *in newborn infants [10]. 75% (30 out of 40) of all neonatal *Pantoea *infections reported in the English literature as of July 2020 occurred in preterm infants [1,2,5,7,11-16]. Most of these infants had at least one comorbid condition. Comorbidities identified in the literature so far include:

1. Prematurity [1]

2. Respiratory distress syndrome [2,7]

3. Patent ductus arteriosus [2]

4. Necrotizing enterocolitis [1]

5. Congenital heart disease [1,7,16]

6. Intrauterine growth restriction [7]

7. Perinatal Asphyxia [17]

8. Prolonged rupture of membranes [11]

9. Chorioamnionitis [15]

10. VACTERL [17].

Source

Outbreaks of *Pantoea* associated bloodstream infections in NICUs were related to contamination of parenteral nutrition [17], intravenous fluid [16], infant formula [18], blood products [19], and anesthetic agents [20]. Tracing the origin of infections that occur without clustering in the NICU is challenging. These are defined as 'sporadic' infections. No conclusive evidence for vertical transmission of *Pantoea* has been documented.

In a single-center retrospective study conducted from 2000-2015 in a tertiary care pediatric hospital in Turkey, the most common specimens from which *Pantoea* was recovered included pus (six specimens, 42.8%), urine (three samples, 21.4%), tracheal aspirate (three specimens, 21.4%), and blood (three specimens, 21.4%) from a total of 15 isolates [9]. In newborn infants, blood is the most common sample from which *Pantoea* is isolated. The usual sources of *Pantoea* bacteremia include central venous catheters and the respiratory tract. Other causes of *Pantoea *bacteremia include urinary tract infections, peritonitis, and skin infections (anterior abdominal wall abscess) [1].

Microbiology

The genus *Pantoea* was created after Gavini et al. in 1989 proposed the name Pantoea agglomerans to include three similar bacteria, namely *Erwinia herbicola, Erwinia milletiae, and Enterobacter agglomerans* showing a high level of genomic relatedness found by DNA hybridization [21]. *Pantoea* is a gram-negative, non-capsulated, non-spore-forming motile bacilli with peritrichous flagella that forms smooth, translucent, and convex colonies in nutrient agar with or without yellow pigmentation. They are facultative anaerobes, oxidase negative. *Pantoea *can utilize D-xylose, D-ribose, maltose, D-galactose, D-mannose, D-fructose, trehalose, and D-mannitol as carbon sources for energy metabolism [21]. There are 20 species in the genus Pantoea comprised of 13 different DNA hybridization groups [22]. *P. agglomerans and P. dispersa *were the first two species identified within the genus. *P. conspicua, P. brenneri, P. septica, and P. eucrina, P. gaviniae, P. calida, P. ananatis *are the other human pathogenic species isolated from various clinical specimens [23,24]. *P. agglomerans* - 95% and *P. dispersa* - 5% constitute the most common species implicated in newborn infants' infections.

Biocontrol Potential of Pantoea

The use of living organisms to control infestations and plant diseases is called biocontrol. Certain *Pantoea *strains grow as epiphytes and have been shown the potential to decrease the incidence of plant disease. In the United States, the nonpathogenic strains of *Pantoea agglomerans* are commercially available and used as a biocontrol agent against plant diseases [25]. *P. agglomerans E325* has been developed as a commercial biocontrol product for use against a variety of plant diseases [26,27].

Pathogenicity

Attempts made to explain the cross-kingdom pathogenicity of* Pantoea spp.* were unsuccessful because the plant and clinical isolates were phylogenetically similar and closely related [28,29]. Recent advances in molecular biology have provided whole genome sequences of several *Pantoea spp. *have helped us reduce the knowledge gap in understanding the determinants of human association of these opportunistic pathogens. The Large Pantoea Plasmid is a candidate plasmid responsible for encoding several proteins that may play a significant role in the colonization, pathogenicity, and antibiotic resistance exhibited by *Pantoea spp.* in the human host [30]. The hcp and vgrG genomic islands present within the type VI secretory system -1(T6SS-1) locus are the other potential evolutionary hot spots responsible for cross-kingdom pathogenesis [31].

Clinical features

The most common presentation of *Pantoea* infections in newborn infants is late-onset sepsis. Outside the NICU outbreak due to contaminated parenteral nutrition reported by Habsah et al. in 2005, there were only two cases of early-onset sepsis so far that has been reported in the neonatal population associated with chorioamnionitis and prolonged rupture of membranes [11,15]. Newborn infants with *Pantoea *bacteremia present with pulmonary symptoms most commonly. The system-wise clinical features of *Pantoea* infections reported in the literature so far are included in Table 1. 

**Table 1:** Clinical manifestations of Pantoea bacteremia in neonates.

System	Clinical presentation
Respiratory	1. Respiratory distress (most common reported presenting symptom), 2. Pneumonia, 3. Pulmonary hemorrhage, 4. Respiratory failure
Cardiac	1. Septicemic shock – requiring vasoactive agents
Neurological	1. Seizures, 2. Intracranial bleed, 3. Temperature instability/fever (100% in one case series)
Hematological	1. Leukopenia, 2. Thrombocytopenia, 3. Disseminated intravascular coagulation (DIC) (87.5% of patients in one case series)
Gastrointestinal	1. Upper gastrointestinal bleeding, 2. Conjugated hyperbilirubinemia
Metabolic	1. Hypoglycemia, 2. Hyperglycemia

Diagnosis

Blood culture and isolation of *Pantoea* is the gold standard in the diagnosis of bloodstream infections. Rapid species-level identification and antibiotic susceptibility are made possible by molecular diagnostic tests like matrix-associated laser desorption/ionization time-of-flight mass spectrometry (MALDI-TOF MS), 16 s RNA gene sequencing, multi-locus sequence analysis (MLSA), cpn60-based typing. *Pantoea* infections are relatively rare but potentially dangerous opportunistic pathogens in NICU, especially in preterm infants, so accurate species-level identification and appropriate directed therapy are essential. Rezzonico et al. compared plant-origin and clinical strains of *Pantoea spp.* in a search for discriminating genotypic/phenotypic markers using multi-locus phylogenetic analysis and fluorescent amplified fragment length polymorphisms fingerprinting. The study found that a large number of clinical isolates from culture collections were found to be improperly designated as* P. agglomerans* after sequence analysis [32].

A recent study used a combination of MLSA and cpn60-based molecular typing of 54 clinical isolates that had been identified as *Pantoea *using MALDI-TOF and other clinical typing methods. They showed that 24% of clinical isolates were misidentified, with MALDI-TOF misidentifying one of every five strains. They found that* P. agglomerans and P. septica *are the two correctly identified species [33].

Management

Antibiotic resistance patterns found in *Pantoea* limits the choice of antibiotics. According to our best knowledge, there are no clinical studies that have compared the antibiotic regimens against Pantoea. The evidence for the efficacy of the various antibiotic choices in neonates is derived from individual case reports and a few case series.

Antibiotic Resistance

*Pantoea agglomerans* have shown resistance to a wide variety of antibiotics, including early-generation penicillin, early-generation cephalosporins, broad-spectrum cephalosporins, and antipseudomonal penicillins, fluoroquinolones, aminoglycoside, TMP-SMX, and tetracyclines.

In a study evaluating powdered infant formula milk samples between 2011 and 2012, *P. agglomerans *were isolated from 6.4% of samples. The isolates showed susceptibility to tigecycline, chloramphenicol, cefepime, levofloxacin, minocycline, and colistin. Fifty percent of the strains in that study were resistant to cefotaxime, moxifloxacin, cotrimoxazole, and ticarcillin [18].

In a case series that included pediatric and newborn infants, all isolates of *P. agglomerans *showed antimicrobial susceptibility to amikacin, gentamicin, meropenem, and trimethoprim-sulfamethoxazole and 92.5% of isolates were susceptible to broad-spectrum cephalosporins and semisynthetic penicillin, 62.3% to extended-spectrum cephalosporins, and only 47.2% to ampicillin [1].

In another study that evaluated the antimicrobial susceptibility of *P. agglomerans* over 15 years in pediatric patients, 21.4% of isolates showed carbapenem resistance [9]. The use of broad-spectrum antibiotics is known to increase the risk of colonization in hospital settings and increase the risk of the emergence of antibiotic resistance. The gastrointestinal colonization and later translocation may act as a reservoir for the organism [34].

Mechanism of Drug Resistance

The drug resistance genes identified in *Pantoea spp.* include CTX-M-15, TEM-1, dfrA14, tet(K), qnrB1, aac-69-16-cr, class1 and class 2 integron, aph, aadA1, cat1, qacDE, Sul1 [35].

CTX-M-15 is the most common, widely prevalent ESBL gene studied concerning the extraintestinal pathogenic *E. coli* ST131 [36]. Amp C beta-lactamase, another important ESBL gene common with *Enterobacter aerogenes*, another important saprophytic gram-negative bacilli, has not been isolated from *Pantoea spp*. Plasmid and integron harbor the candidate genes involved in drug resistance associated with *Pantoea spp*.

Treatment

Success in managing *Pantoea* bloodstream infections in newborn infants depends on prompt identification and early administration of appropriate antibiotic therapy combined with routine supportive care. Since most of the reported Pantoea spp. BSI in newborns has been associated with central venous lines; care should be taken to identify this association. 

Antimicrobial selection

First-Line Therapy

We suggest that the neonatologist pay attention to the site of infection, gestational age of the infant, and their comorbidities while choosing the antimicrobial treatment. For term or late preterm infants with infections occurring without central venous lines or other comorbidities, the aminoglycoside (gentamicin or amikacin) combined with ampicillin is the appropriate first-line therapy [15].

In a case series that had reported positive outcomes in very preterm infants with *Pantoea* BSI in the presence of central venous lines and comorbidities with or without hemodynamic comprise, broad-spectrum antibiotics like a combination of third-generation cephalosporin with aminoglycosides or carbapenems or ureidopenicillin/ beta-lactamase were used as the first line of therapy [2]. 

Based on our experience (unpublished data) with this organism as an etiological agent causing late-onset sepsis in extremely preterm infants with comorbidities (ventilator-dependent bronchopulmonary dysplasia and central venous catheter), we recommend carbapenems as first-line therapy for pneumonia or bacteremia. For uncomplicated infections limited to the genitourinary tract, aminoglycosides could be used as the first-line therapy.

Alternative Therapy

For *Pantoea* strains resistant to carbapenems, trimethoprim/ sulfamethoxazole can be a good alternative. This medication in neonates requires regular monitoring of serum bilirubin levels and liver enzymes during the entire course of therapy.

Prognosis

*Pantoea *bloodstream infection being an opportunistic pathogen affecting infants with comorbidities, has a high mortality rate. The mortality rate is exceptionally high in preterm infants. The reported mortality rate among the case series and case reports of *Pantoea* infections in the NICU is 45% (18/40). More than 95% of those infants were premature at birth. The most common cause of mortality is respiratory failure and septic shock. In one case series, including five preterm infants with *Pantoea* BSI who were started on meropenem or piperacillin/tazobactam as first-line antibiotics had a favorable outcome of survival in all of them [2]. On the contrary, the exceptionally high mortality rates of 87.5% (7/8) [17] and 100% (3/3) [7] have been reported in case series describing *Pantoea *outbreaks in NICUs.

## Conclusions

*Pantoea *is a relatively rarer but potentially dangerous hospital-acquired infection encountered in NICUs, especially in preterm infants with comorbid conditions. *P. agglomerans and P. dispersa* are the most common pathogenic species in newborns. Colonization tends to happen in the respiratory tract, genitourinary tract, and gastrointestinal tract. Translocation into the bloodstream produces aggressive infection. Sensitivity to routine first-line antibiotics is variable with a high prevalence of multidrug resistance. In this scenario, neonatologists should clearly understand this pathogen, which would help treat the neonates with this infection efficiently. With limited available literature, multicenter data collection may facilitate more extensive studies that could yield valuable information to assist in the clinical management of *Pantoea* infections.
